# Evaluating Patients’ Experiences with Healthcare Services: Extracting Domain and Language-Specific Information from Free-Text Narratives

**DOI:** 10.3390/ijerph191610182

**Published:** 2022-08-17

**Authors:** Barbara Jacennik, Emilia Zawadzka-Gosk, Joaquim Paulo Moreira, Wojciech Michał Glinkowski

**Affiliations:** 1 Polish Telemedicine and eHealth Society, 03-728 Warsaw, Poland; 2 Multimedia Department, Polish-Japanese Academy of Information Technology, 02-008 Warsaw, Poland; 3 International Healthcare Management Research and Development Center (IHM-RDC), Shandong Provincial Qianfoshan Hospital, Jinan 250014, China; 4 Gestao em Saude, Atlantica Instituto Universitario, 2730-036 Oeiras, Portugal; 5 Center of Excellence “TeleOrto” for Telediagnostics and Treatment of Disorders and Injuries of the Locomotor System, Department of Medical Informatics and Telemedicine, Medical University of Warsaw, 00-581 Warsaw, Poland

**Keywords:** free-text narratives, information extraction, linguistic analysis, patient experience, text mining, technological innovation

## Abstract

Evaluating patients’ experience and satisfaction often calls for analyses of free-text data. Language and domain-specific information extraction can reduce costly manual preprocessing and enable the analysis of extensive collections of experience-based narratives. The research aims were to (1) elicit free-text narratives about experiences with health services of international students in Poland, (2) develop domain- and language-specific algorithms for the extraction of information relevant for the evaluation of quality and safety of health services, and (3) test the performance of information extraction algorithms’ on questions about the patients’ experiences with health services. The materials were free-text narratives about health clinic encounters produced by English-speaking foreigners recalling their experiences (*n* = 104) in healthcare facilities in Poland. A linguistic analysis of the text collection led to constructing a semantic–syntactic lexicon and a set of lexical-syntactic frames. These were further used to develop rule-based information extraction algorithms in the form of Python scripts. The extraction algorithms generated text classifications according to predefined queries. In addition, the narratives were classified by human readers. The algorithm-based and the human readers’ classifications were highly correlated and significant (*p* < 0.01), indicating an excellent performance of the automatic query algorithms. The study results demonstrate that domain-specific and language-specific information extraction from free-text narratives can be used as an efficient and low-cost method for evaluating patient experiences and satisfaction with health services and built into software solutions for the quality evaluation in health care.

## 1. Introduction

Text databases often contain unstructured or semi-structured data. Repositories of verbal content, such as free-form texts, interviews, survey responses, medical documentation, or health care unit communications, are valuable data sources for research in the health services and public health sectors [1,2,3,4]. The applications for information extraction from clinical text data have been developed for at least a decade [5,6,7]. The study was inspired by research focused on patients’ experience and satisfaction and spanned the boundaries of a few disciplines [8]. The most advanced healthcare systems usually include patient experience and satisfaction measures in their service delivery models. Modern healthcare management solutions are increasingly focused on patient-centered care, optimizing patient clinical outcomes and system performance [9,10]. It is also parallel to narrative-based medicine (NBM), which postulates the need for the patient to tell their own story and for the clinicians to understand the patients’ stories.

“NBM shifts the doctor’s focus from the need to problem-solve to the need to understand. As a result, the patient-physician relationship is strengthened, and the patient’s needs and concerns are addressed more effectively and with improved health outcomes” [11]. The patients’ free-text narratives about their experiences improve their engagement in the treatment process. The narrative-based medicine approach is practiced in some health systems [12,13]. The patients’ experience narratives may be collected as data and analyzed by health services providers for research and clinical purposes, such as clinical outcomes, adverse drug effects, and health system effectiveness [14,15,16]. Both factors can improve services, reduce costs, and improve patient self-management. Greater efficiency of these services can also improve access to healthcare for underdeveloped populations. Health systems evolve, as do the development of healthcare infrastructure and technology [17]. The understanding of the importance of digital technologies in healthcare systems has increased among clinicians. Healthcare administration should use intelligent systems that can improve medical history by storing the history in more extensive databases and thereby provide better treatment.

This article shows how patient narratives can be used to extract meaningful information on the effectiveness of health services. Other research and clinical applications of free-form written and spoken texts can be envisaged, including patient complaints assessments or interviews before or after the face-to-face or virtual visit. The methodological approach may lead to the development of applications that can improve the duration and effectiveness of the medical interview during the patient’s visits.

In various service sectors (e.g., health, education, etc.), large volumes of unstructured texts are collected and used for research [18,19,20,21]. The manual coding techniques and information retrieval methods are labor-consuming. Instead, automatic text-mining techniques can be used as an alternative. The availability of large volumes of data accessible online or in one text repository has driven a dynamic development of text-mining and information-extraction methodologies for processing of free-form texts [5,22]. The methods may focus on keyword search, entity recognition, event identification, relationship analysis, and other tasks [23]. Rule-based techniques [24] are the classical solution that could be applied to smaller amounts of data and usually do not need sophisticated data preprocessing or model training. The different data-mining methods, such as association rule mining [25] or short-texts clustering [26,27], became popular as more data were generated. The development of Bayesian methods [28] and machine learning techniques entailed their application to information extraction [29] or opinion sentiment analysis [30]. 

The main aims of this research were to (1) elicit patients and their companions’ free-text narratives about the experiences of English-speaking foreigners with health service encounters in the healthcare facilities in Poland, (2) develop domain-specific algorithms of information extraction and test their performance on a collection of elicited narratives, and (3) answer selected questions about the foreigners’ experiences and satisfaction with health services in Poland.

## 2. Materials and Methods

### 2.1. Participants and Materials

The study participants were international students in a master’s degree program in business management at a university in the Mazovia region of Poland. Altogether, 111 individuals were invited to participate in the study, and 104 participants filled out the response packets according to instructions and were included in the analysis. The response packets contained questions concerning age, nationality, gender, and duration of stay in Poland. There were a greater number of males (*n* = 80; 76.9%) and fewer female participants (*n* = 24; 23.1%). The majority were from India (*n* = 96), a few from Nepal (*n* = 7), and one from central Asia. Their age range was 18 to 34 years. At the time of data collection, the participants stayed in Poland for between 4 months to 3 years. They were all enrolled in an English-language master’s level program and communicated primarily in English at the university and in work settings in Poland.

### 2.2. Ethics Approval

The study was conducted according to the guidelines of the Declaration of Helsinki and approved by the Scientific Research Committee of the University of Euroregional Economy in Józefów, Poland (letter of approval obtained on 16 December 2020). Informed consent for participation was brought before the study from all respondents. The aim of the study was explained orally and in writing on the response packet. In addition, the participants manually signed a list of participation as proof of their informed consent to participate. The lists of participants’ signatures were kept separately from the response packets, so the individual response packets remained anonymous. The participants’ sensitive data were not collected. The response packets did not contain any personal identification questions. The data subjected to analysis did not include any personal or organizational identifiers. Thus, anonymity of the respondents and organizations could be guaranteed.

### 2.3. Characteristics of Texts and Preprocessing of Textual Data

In their response packets, the respondents produced short stories describing their experiences with the healthcare facilities in Poland. There were 104 stories included in the analysis. There were 45 first-person accounts of encounters at medical clinics and hospitals and 59 reports of patient experiences told by patients’ close friends or relatives who accompanied a patient when seeking medical help. All stories were written in English.

Initially, all texts were handwritten by the participants within the response packets. The stories were later dictated voice-to-text by a near-native English speaker using the Microsoft Office 365 voice recognition [31] functionality and converted to a text-only utf-8 format for further analysis. During dictation, some necessary editing of texts was performed. Any identifying information was removed if a respondent disregarded instructions and signed a response packet. For words that were illegible and impossible to identify from context, the letter string “xxx” was substituted. Any evident spelling mistakes were corrected during dictation. The texts were segmented into sentences marked with a capital letter at the beginning of each sentence and a period in the end. The original sentence segmentation by the respondents was preserved. When respondents left out segmentation marks in locations where a terminable sentence unit was evident based on the semantic context, the missing segmentation marks were added (period, space, capital letter).

### 2.4. Research Questions about Patients’ Experiences

The general research problem addressed in the study were patients’ experiences during health clinic encounters and, indirectly, the health care system’s effectiveness in serving foreign patients seeking medical help.

Our investigation focused on the extraction from free-text narratives of information pertaining to selected topics within the patient’s experiences. Four aspects of patients’ experience and satisfaction were selected, based on the preliminary exploration of the text collection, as the most salient in the patients’ stories. The following four topics were chosen as the focus of investigation:

- Language-communication difficulties experienced by patients during interaction with healthcare unit personnel;
- Type of visit to the healthcare facility—seeking medical checkup vs. treatment for illness or injury;
- Type of medical personnel involved in the provision of health services to patients;
- The number of healthcare facilities patients visit on one occasion of seeking treatment.

### 2.5. Development of the Lexicon and Lexical–Syntactic Frames for the Automatic Query of Texts

The lexicon and the lexical–syntactic frames for this study were developed using an experimental software Text Analysis AT [32], designed and developed as part of an earlier project (2011). It is an offline program for searching and counting user-defined terms and phrase/sentence templates (frames). The result of the predefined searches are lists of tokens or types of phrases and sentences. The program allows the user to define lexicons or categories of searched words. It allows one to search for sentence patterns by pointing to user-defined morphological structures of words. The program can be adapted to inflectional languages [33].

The automatic search for key critical elements and classification of texts required the formulation of rules for the semantic–syntactic lexicon and lexical–syntactic phrase and sentence frames. The automated information-extraction tasks usually require constructing ad hoc lexicons relevant to a given speaker population and situational knowledge. The lexicon for this study was built as a set of word groups from the collection of texts (Supplementary Files S1). This lexicon is specific to a particular collection of texts, a given population of English language speakers, and a defined set of events or situations.

The word groups in the lexicon were composed of wordforms (defined as inflected lexemes) of the same semantic category and usually of the same syntactic class (part of speech). The classification into word groups was not exclusive. For example, the word “medical” was assigned to the class of adjectives and the class of nouns. The first step in the lexicon design process was a type/token search that produced frequency lists (Supplementary Files S1).

In the next step, word groups differing in syntactic functions were determined, and the function and content words were separated. Function words are restricted in number and occur with high frequency. They are often referred to as closed-class words. The content words are numerous and occur with lower frequency. Once the function words were identified on the frequency list, they were filtered out by setting the filter to a minimum of 4 letters. This procedure allowed us to remove most of the high-frequency function words. Next, the words carrying meanings relevant to the study topics were selected on the shortened list. Only the words with potential reference to key issues were classified into semantically and syntactically defined word groups. The classification of wordforms into word groups assumed that a wordform could be assigned to more than one semantic-syntactic category.

The relationships between word groups varied. The groups could be (a) independent relative to each other (no wordforms belonging to both), (b) overlapping, or (c) superordinate/subordinate. The latter can be exemplified with a relationship of the group “event noun” that is superordinate to the group “event negative noun”. A further assumption was made that the lexicon should be minimal, containing the smallest number of semantic–syntactic word groups necessary to compose lexical–syntactic frames representing the critical statements. More examples of word groups are in two tables in Supplementary Files S2.

Along with the process of defining the necessary word groups, the texts were searched for key statements relevant to the four research topics on health care. The individual statements were used to construct more general lexical–syntactic frames (Supplementary Files S2). Next, the searches were performed using the Text Analysis program to test the validity of each lexical–syntactic frame as an information retrieval tool for a given query. A lexical–syntactic frame is defined as a series of constituents comprised of words, or categories of words, bound in an ordered sequence. Two types of constituent binding within a frame were defined: (1) adjacent sequence (symbol ++) when two constituents occur immediately one after another and (2) within-sentence sequence (symbol +) when two constituents occur one after another within the same sentence, being adjacent or not. Examples of lexical–syntactic frames are presented in Supplementary Files S3.

### 2.6. Development of Algorithmic Rules for Automatic Queries of Texts

A paradigm shift in natural language processing research has been observed in recent years [34,35]. The rule-based approach to information extraction tends to be abandoned in favor of the Bayesian methods [28] and machine learning techniques [36,37] and the deep learning/deep neural networks (DL/DNNs) [38]. Bayesian models, due to their operating principle, which is based on probabilities and the learning method, require large amounts of data and considerable computational resources to be able to provide answers with satisfactory precision. Their successors, DNNs are synchronizable to achieve high performance while saving time and resources on time-consuming manual feature selection [38]. However, constructing a DNNs system requires even larger amounts of data for iterative training cycles and greater computing resources. Creating a simple rule-based system may be a better choice depending on the research problem being addressed and the type of text corpus used. It is especially the case for texts addressing a narrowly defined topic and written in a unique sociolinguistic style. With smaller text corpora, simpler methods may be more advantageous both in terms of effort, time, and computing power needed as well as the quality of the expected results. All the calculations performed in our solution required very little computing resources and were performed on a popular and widely available computing platform.

The rules-based approach to information extraction was adopted [24]. The main task performed by the algorithms was to identify the keyword strings generated from predefined lexical–syntactic frames and a domain-specific semantic–syntactic lexicon. The word sequences/strings corresponded to the queries of facts and opinions.

The solution for automatic responding to queries was created as a set of scripts implemented according to the described lexical–syntactic frames/patterns. The query rules were established as the series of direct and indirect neighborhoods of words grouped into function words and content words classes. The categories were prepared as comma-separated text files, which allow easy word lists modification. The scripts were created in Python using spaCy [39] and Pandas [40] modules. SpaCy is an advanced natural language processing library that supports a fast and efficient way of analyzing unstructured textual data. The program was developed using the library’s rule-based matching engine. The engine is designed for easy searching for words and patterns in texts. Respondents’ free-text files were converted to utf-8 format and read by the scripts. Firstly, the content was preprocessed using the SpaCy language model [41], including tokenization and part-of-speech tagging. A separate program was created for each research question, which returns an answer based on the implemented rules. Queries Q3a and Q3b were included in one script.

Different categories of words are represented as array structures from which the search word can be selected and checked in the analyzed context. As determined in a matching pattern, the sequence of words can be adjacent or within-sentence. Within-sentence neighborhood means that there can be any number of series consisting of alphabetic characters that are not separated by a sentence segmentation mark between the specified words. As the period is not included in the matching rules, the pattern is always searched for within the same sentence. Found patterns are added to the Pandas data frame with the label of returned answer. When the script has classified all texts, a comma-separated file is created. The results returned by the programs were tested by comparison with the results of human evaluation and semi-automatic pattern search using our Text Analysis software. The final version of the code is available in the Google Colab repository [42].

### 2.7. Formulation of Natural Language Questions for Human Readers’ Query of Texts

In addition to the automatic information extraction, a method of human reader evaluation of text content was used to obtain data for benchmarking. Two human readers read all 104 texts independently. The readers’ task was to answer questions corresponding to four patient experience topics under investigation. For the human readers’ query of texts to match the automatic rule-based queries, the questions for readers were formulated as five closed-ended dichotomous questions. In the case of topic 3, “Type of medical personnel involved in the provision of health services to patients”, this required formulating two questions. The questions for human readers’ query of texts were as follows:

- Q1. Does the text mention any difficulties in language communication?
- Q2. Does the text mention illness or injury as a reason for contact with a healthcare facility?
- Q3a. Does the text mention any physicians or physicians involved in providing health services?
- Q3b. Does the text mention personnel other than physicians involved in providing health services?
- Q4. Does the text mention seeking medical help in more than one healthcare facility?

## 3. Results

### 3.1. Statistics for the Text Collection

Speech-to-text dictation of handwritten texts was possible thanks to the legible writing style of most respondents. (In India, good handwriting is required in the educational system, and students need to know at least two writing scripts.) Only a tiny percentage of texts were rejected as mostly illegible (*n* = 2). There were instances of individual illegible words in the remaining texts that did not affect the understanding of texts. It took 1–5 min to dictate one story, including editing the errors of dictation, spelling, or punctuation. Out of 111 response packets completed by the respondents, only seven stories were rejected; the exclusion was due to illegibility (*n* = 2) or inconsistency with the story-writing instructions (*n* = 5). A total of 104 texts were legible and consistent with the instructions and were included in the analysis. The following descriptive statistics for the text collection are presented in Table 1: total number of texts, total number of sentences, mean number of sentences per text, minimum and maximum number of sentences in a text, total number of wordforms (tokens), mean number of wordforms (tokens) per text, minimum and maximum number of wordforms (tokens) in a text, total number of different wordforms (types), mean number of different wordforms (types) per text, and minimum and maximum number of different wordforms (types) in a text.

The participants’ stories were composed of syntactically simplified sentences that often contained grammatical mistakes. However, the accounts of medical encounters were fully comprehensible and conveyed most of the facts and opinions related to patients’ experiences in the health care system. Independently of culture, syntactic, vocabulary, and spelling errors are common in written texts produced by people who are not trained in professional writing. These errors occur in texts whether first- or second-language speakers write them. The automated processing of natural language texts must consider the grammatical imperfections of texts generated by non-professionals or second-language speakers. In this text collection, in most cases, the grammatical errors did not interfere with understanding the key elements of meaning.

### 3.2. Results of the Python Script Classifications

The respondents’ texts were searched (queried) with Python script algorithms written based on inputs from the semantic–syntactic lexicon and lexical–syntactic frames developed for the study. For each of the five queries on topics related to patient experience (language communication, medical personnel involvement, facility change, and type of medical service), when the Python script pattern search returned at least one positive result, the text was attributed score “1”, and when searching for patterns gave a negative result, the text was attributed score “0”. The results of classifications were in the form of an Excel data file. The classifications of texts performed by the Python script were summed up for each question for the whole collection of texts and are presented in the last two columns of Table 2 as absolute numbers and as percentages of the total number of texts (*n* = 104). The data in columns 1–4 of Table 2 contain human readers’ classifications summed up for each question in absolute numbers and as percentages of the total number of texts.

### 3.3. Results of Human Readers’ Classifications

The results of the classification of texts by two human readers were highly consistent. The inter-rater agreement for the five questions was, respectively: 100%; 92.3%; 98.1%; 69.2%; 98.1%. However, the reader’s responses were not fully in agreement, which is not surprising given that people vary in their understanding and interpretation of texts. To accommodate this slight variability, the two readers’ responses to questions were converted to a series of rank classifications of texts. For each of five questions, the texts were rated for containing an answer to the question. The rank score for a text was “1” when both readers answered yes; when one reader answered yes and another no, the text score was “0.5”; when both readers answered no, the score was “0”. The classifications of texts performed by the human readers were summed up for each question for the whole collection of texts and are presented in Table 2.

### 3.4. Results for Correlations of the Automatic and Human Reader Classifications

The classifications of texts by human readers were represented as single variables based on the rankings of consistency between two human readers. The Python script scores automatic classifications and the single-variable human reader classifications were correlated pairwise for each of five questions using the Spearman rank correlation coefficient. The correlations were all significant (*p* < 0.01), with four questions indicating high correlations (Table 3) [43]. The calculations were performed using the IBM SPSS [44] Statistics 25 software.

Both the Python script and human readers’ classifications provided valuable information regarding aspects of patients’ experience and satisfaction: (1) communication difficulties, (2) type of healthcare service provided, (3) involvement of physicians, (4) involvement of other health service personnel, and (5) seeking medical help in more than one healthcare facility (Table 2). The automated and human readers’ queries of the content of free-text narratives were highly correlated and significant (Table 3).

## 4. Discussion

An important motivation of our research is the observation that text mining has often been used to extract information from the electronic medical records, including free-text descriptions produced by professionals. On the other hand, text mining is rarely applied to extract information from free-text narratives of the patients or the nonpaid patient caregivers. The experiences and satisfaction of patients and their nonpaid carers with health care services are usually evaluated using other types of techniques, e.g., closed-ended item surveys or qualitative interviews. Both methods have limitations, such as potential research bias, difficulty recruiting respondents, complexity of results, and cost of the preparation and running of a study. Therefore, an important motivation of our study was to demonstrate the effectiveness of the extraction of information from free-text patients’ experience stories. We believe that this approach is highly promising and deserves greater attention of the researchers specializing in text mining methods as well as those focused on health service evaluation.

The study found that algorithmic and human text classification results were very similar in response to questions about the four aspects of experience and patient satisfaction. Only the query about non-physician medical personnel was performed by the algorithm less accurately than were the answers provided by the human readers. The likely reason why this one query was less effective is that the narrators tended to describe non-physician medical personnel with backgrounding expressions, such as third person plural pronouns (“they”), and tended to establish reference using nouns with broader meaning (e.g., “lady”).

The knowledge schemas and story scripts underlying the patients’ story construction were relatively simple. The actors, locations, actions, and events mentioned in the stories were limited, and the relationships between different entities were not complex. Generally, it can be argued that the scripts of stories about patient experiences in medical encounters and their underlying ontology are relatively universal. They can be described as a family of specialized scripts that can be classified according to the critical groups of variables—demographic, geographic, and health service specialization. A library of story scripts of medical encounters would be a valuable resource for the developers of algorithms for querying texts.

Necessary conditions facing any research conducted with patients’ data include the stipulation to ensure anonymity by removing any personally identifying information and, whenever applicable, to obtain consent to participate. When these requirements are met, and the appropriate anonymization and consent procedures are followed, the nonclinical free-text narratives can be efficiently elicited from former patients, their companions, and carers and subjected to analysis using text-query algorithms. Such data obtained from natural text or transcribed speech can be a rich source of information more diverse and valid than the traditional closed-ended items questionnaires. Free-text narratives can be mined for information about various aspects of the health system’s operation: a demand for health services, accessibility of services, patient opinions, and satisfaction. The free-text narratives about healthcare encounters can provide direct insights into factors determining patients’ overall satisfaction with health services. In addition, narrative text data can be used to measure the level of overall satisfaction with health services, which can be expressed in patients’ stories with various text-stylistic features covered by the notion of sentiment. The sentiment indices of texts can be studied using a rule-based sentiment analysis [45] or an automatic approach using language models such as GPT [46] or BERT [47]. The next stage of this project is to focus on developing algorithms precisely measuring patients’ satisfaction from a collection of free-text narratives.

The presented rule-based approach to information extraction is different from a typical machine learning approach. We did not divide the collection of stories into three separate data sets: training, validating, and testing. The same set of texts was used to construct the lexical–syntactic rules that fed the query algorithms and was used for the human readers’ query and classification of texts. Therefore, from the machine learning perspective, our algorithms went only as far as the training and, perhaps, validation phase and were not yet tested on a separate testing data set.

However, it can be argued that patient stories are only universal to a limited extent, and individual realizations vary considerably depending on linguistic, geographic, demographic, clinical groups, healthcare, and procedures. Therefore, the usual machine learning approach of building predictive models based on diverse large volumes of data is not the most effective for analyzing patients’ experience stories. Conversely, working with homogeneous collections of patients’ narratives and utilizing multiple metadata information can be equally effective.

Our study included a medium-sized group of participants (*n* = 104) chosen because of their homogeneity in terms of the language output. We believe that this is an advantage of the study, and it can be argued that the heterogeneity of groups of participants may be a drawback of text-mining research on large text corpora, which are often missing relevant information on demographic and contextual characteristics. The results of our study demonstrate how localized background knowledge of patients’ experience and of the linguistic structures of language can be used effectively to develop algorithms using available programming tools (e.g., Python) that can be applied to accessible and inexpensive data source of free-text narratives with good results.

The conducted research has shown that with minimal preparation of textual data (only spelling correction and segmentation into sentences), it is possible to construct domain-specific grammatical rules, which have turned into algorithms that can be applied to texts of any form, not edited for syntax and vocabulary imperfections, and to classify these texts in a way very similar to the classification of human readers in response to equivalent natural language queries.

It can be argued that applying domain- and language-specific rules designed to respond to narrowly defined queries can be as effective as human readers’ classification of texts and be performed at a much lower cost. In general, information extraction from the homogenous collection of patients’ free-form texts tagged with extensive metadata information can be an effective method for evaluating patients’ experiences and, ultimately, for the health service evaluation.

Given the diversity of clinical populations as well as of the health services provision, the evaluations of health services are rarely representative of all the clinical and demographic groups and types of services. It is worth noting that a health care unit rarely provides services to a whole demographic spectrum of population. Specialization with respect to population segments appears to be a norm and not a rule.

The text-mining results obtained in the study are representative for the particular ethnic group in a particular healthcare provision situation, i.e., the South Asian students using health services in Poland. This is not an atypical population segment receiving healthcare services in Poland. The health facilities infrequently deal with health needs of this demographic group, ethnically and linguistically non-Polish, speaking a variety of English not commonly spoken by the health service providers in Poland.

The selection of a homogenous group of patient narrators was intentional because the extraction of information from texts is most effective and reliable when the texts are similar with respect to: text genre, characteristics of text producers, addressees/audiences, functions of the discourse, and time and space localization of texts. A different segment of foreign patients in Poland using a different variety of English or of different age groups or educational statuses would likely produce a set of very different narrative texts in terms of the lexical, syntactic, and discourse characteristics and would narrate about a different set of medical events, healthcare facilities, and types of treatment.

An important point we wish to make in this paper is that free-text narrative data related to patient experiences can be analyzed most effectively when the text corpus can be subdivided along the demographic and medical context features. We believe that the approach we used could be applied universally.

Our study could be seen as a pilot for evaluation of health services provided to a specific population segment. The evaluation based on a collection of free-text narratives only applies to a given demographic population segment and type of health service. For full-scale evaluation, the number of narratives would need to be increased and more variables related to the demographics and context embedded in the study design.

## 5. Conclusions

The domain- and language-specific information extraction from free-text narratives is a promising methodology for discovering issues and concerns frequently reported by patients. Carefully created searching patterns can return results close to the performance of human readers’ extraction of information from free-form texts. During the recent COVID-19 pandemic [48], millions turned to social media to tell their personal stories of illness and recovery. This unprecedented wave of expressive writing resulted in massive amounts of free-form texts available for analysis and development of text-mining and information-extraction tools. The presented study started before the COVID-19 pandemic came to Europe, and we could not have predicted that our results could be applied to free-form texts about the pandemic.

Summing up, the following main results of the research carried out should be highlighted: (1) Tools were developed to extract information from a collection of free-text patient experience stories; (2) the research results have shown how automated free-narrative analysis can be used to answer some questions about the functioning of a health system, in particular, the patients’ experiences during encounters in healthcare facilities; and (3) the study showed that domain- and language-specific unstructured text corpora could be efficiently explored and searched to find relevant information using rule-based information-extraction algorithms.

The study shows a promising direction for future research. Once the technological and anonymity assurance issues are fully resolved, extensive collections of free-text narratives describing patients’ experiences can become a rich data source on health services’ quality, safety, and effectiveness. The presented approach can further develop low-cost health service evaluation applications and improve health care systems.

## Figures and Tables

**Table 1:** Statistics for the text collection.

Unit of Analysis	N (Total)	Mean (in Text)	Min (in Text)	Max (in Text)
Texts	104			
Sentences	958	9.21	3	23
Wordform tokens	13,632	131.08	29	279
Wordform types	1445	13.89		

**Table 2:** Human readers’ and Python script classifications for five questions on patients’ experience for the collection of texts (*n* = 104).

Questions	Reader 1	%	Reader 2	%	Readers’ Mean	%	Python Script	%
Q1. Communication	41	39.4	41	39.4	41	39.4	43	41.3
Q2. Type of Service	87	83.7	81	77.9	84	80.8	79	76.0
Q3a. Physicians	80	76.9	80	76.9	80	76.9	80	76.9
Q3b. Other Personnel	55	52.9	65	62.5	60	57.7	37	35.6
Q4. Change of HCU	13	12.5	11	10.6	12	11.5	9	8.7

**Table 3:** Correlations of Python script and single-variable ranked readers’ classifications for five questions on patients’ experience.

No.	Questions on Patients’ Experience	Rho Spearman Rank Coefficient
Q1.	Does the text mention any difficulties in language communication?	0.881 **
Q2.	Does the text mention illness or injury as a reason for contact with a healthcare facility?	0.921 **
Q3a.	Does the text mention any physician or physicians involved in providing health services?	0.941 **
Q3b.	Does the text mention personnel other than a doctor who is involved in providing health services?	0.511 **
Q4.	Does the text mention seeking medical help in more than one healthcare facility?	0.831 **

** *p* < 0.01.

## Data Availability

Part of the data presented in this study are available in Supplementary Files: Supplementary Files S1: Wordform types and tokens; Supplementary Files S2: Semantic–syntactic word groups lexicon. Excel worksheets: Function words, Content words; Supplementary Files S3: Lexical–syntactic frames. Information and text samples from the collection of patient experience stories are available on request from the principal author, B.J. The data are not publicly available due to privacy reasons.

## Supplementary Materials

The following supporting information can be downloaded at: https://www.mdpi.com/article/10.3390/ijerph191610182/s1, Supplementary Files S1: Wordform types and tokens; Supplementary Files S2: Semantic–syntactic word groups lexicon. Excel worksheets: Function words, Content words; Supplementary Files S3: Lexical–syntactic frames.
