# Supplementary material for: Evaluating Patients’ Experiences with Healthcare Services: Extracting Domain and Language-Specific Information from Free-Text Narratives
Source: Int J Environ Res Public Health. 2022 Aug 17;19(16):10182. doi: 10.3390/ijerph191610182 (PMC9408527; doi:10.3390/ijerph191610182)
Supplement: Supplementary file 1 [file ijerph-19-10182-s001.zip › Supplementary Files S2.pdf]

Supplementary Files S2

Semantic-syntactic word groups lexicon part 1 - function words

ArtDemSpec	ChangeSpec	OrdNum	CardNum	DigitNum	BeV	DoV	CanV	ShallV
a	other	First	one	1	be	do	can	shall
an	another	second	two	2	I'm	does	could	should
the	previous	third	three	3	am	did	couldn't	shouldn’t
this	next	1st	four	4	is	done		
that		2nd	five	5	are	doesn't		
these		3rd	six	6	was			
those			seven	7	were			
some			eight	8	isn’t			
no			nine	9	aren’t			
next			ten	10	wasn’t			
another					weren’t			
other								
else								
previous								
last								
same								

ShallV	GetV	HaveV	Conj	ClauseConj	VNeg	SubjPro	ObjPro
shall	get	have	and	because	don't	I	me
should	got	Has	then	if	doesn't	you	you
shouldn’t		hasn't	next	so	not	she	her
		Had	but	later	can't	he	him
		hadn't	so	though	couldn't	it	us
			as	but	didn’t	we	them
			also	either	wasn’t	they	
			later				
			though				
			if				
			or				
			because				
			either				

NegPro	PosPro	QuantifPro	ClausePro	Prep	SentAdv	Intensif	Quantif
no one	my	everything	that	in	also	very	only
nothing	your	everybody	who	to	actually	a lot	many
nobody	his	each	which	of	soon	enough	more
	her	anyone	where	on	there		any
	our		when	up	even		all
	their		what	down	finally		each
			once	about	further		most
			since	for	again		every
			while	with	eventually		whole
			whom	from	suddenly		few
				after	of course		a lack of
				before			
				near			
				out			
				at			
				by			
				over			
				along			
				around			
				o			

QuantifZero	QuantifSing	QuantifPlural	QuantifMax
no	only	many	all
a lack of	any	more	most
	each	Few	every
			whole

Semantic-syntactic word groups lexicon part 2 - content words

Adj	Adv	KnowV	EventN	EventNegN	LangComN	LangComV	LangComContV	HCUn
useful	also	know	difficulty	difficulty	call	call	communicate	hospital
good	actually	knows	difficulties	difficulties	communication	communicate	explained	clinic
kind	soon	knew	experience	problem	contact	contact	speak	emergency
careful	once		experiences	problems	conversation	explained	speaking	apteka
	there		problem	issue	English	informed	talk	clinicme
	even		problems	issues	language	said	understand	laboratory
	thoroughly		issue	trouble	Polish	screaming	understanding	ambulance
	finally		issues	troubles	response	speak		hosp
	further		trouble		talk	speaking		hospitals
	tomorrow		troubles			talk		cliniclu
	carefully					tell		hosphospita
	well					told		clinical
	again					translate		Hospital by
	enough					understand		szpital
	eventually					understanding		hospitalname
	suddenly							hospital
	of course							clinicat
								ochotal

HCUnType	InsurPaym	DocumFormN	AccompPerson	MedPersonN	MedPersonDocN	MedPersonOtherN	MedV	MedTreat
hospital	Insurance	visa	friend	doctor	doctor	staff	consult	tests
clinic	Money	document	friends	doctors	doctors	reception	treat	reports
emergency	pln	documentation	roommate	staff	specialist	receptionist	checkup	testing
apteka	visa	forms	brother	reception	physician	person	examine	examination
laboratory	free	identification	colleagues	receptionist	orthopedist	nurses	diagnose	consultation
ambulance	fees	passport	husband	person	dr	stuff		medicines
hospitals	bill		roommates	nurses				results
	covered		colleague	stuff				operation
	insurer		cousin	specialist				prescription
	zloty			physician				medicals
	costly			orthopedist				antibiotic
	payback			dr				temperature
	payment							tube
	fee							examinations
	coverage							prescribed
	account							tablets
	charges							pressure
	paying							examine
	refunded							stitch
	cash							b
								diagnose
								medications

MedTreatObjectN	PatientBodyOrgan	PatientCondition	PatientIllness	PatientBodyFunct	PatientFeeling	PatientEventAtHCU
test	heart	accident	accident	blood	shivering	appointment
medicine	hands	afraid	bleeding	urine	screaming	visit
report	elbow	bleeding	broke	bleeding	sick	admittance
tests	bones	blood	cold	swollen	headache	hospitalization
reports	ligaments	breath	cracked	fever	pain	consultation
medicines	feet	broke	disease	stool	suffering	emergency
results	tongue	broken	dislocated	weight	suffered	
prescription	lungs	cold	disorder	height	painful	
antibiotic	bone	cough	emergency	pressure	scared	
tube	tonsil	cracked	fever	vomiting	shocked	
tablets	nose	disease	headache	breath	tensed	
stitch	eye	dislocated	hernia	cough	nervous	
medications	wrist	disorder	hurt	shivering	nervousness	
sample	arm	emergency	illness	screaming	afraid	
painkillers	eyes	fever	infection		suffer	
stitches	neck	fever	injured		pained	
sonography	kidney	headache	injury		weakness	
medication	knee	height	malaria			
cream	teeth	hernia	pain			
glucose	skin	hurt	poison			
bottle	hand	illness	pregnant			
tablet	stomach	infection	sick			
injection	ear	injured	stone			
cotton	back	injury	swollen			
stick	leg	malaria	tumor			
drops	blood	nervous	viral			
prescriptions	face	nervousness	weakness			
diet		pain	ache			
tubes		pained	appendicitis			

painful
poison
pregnant
pressure
scared
screaming
shivering
shocked
sick
stone
stool
suffer
suffered
suffering
swollen
tensed
tumor
urine
viral
vomiting
weakness
weight
allergic
allergy
allergies
ache
appendicitis
diabetes
ill
bump
pimple

diabetes
ill
bump
pimple
