# Supplementary material for: Evaluating Patients’ Experiences with Healthcare Services: Extracting Domain and Language-Specific Information from Free-Text Narratives
Source: Int J Environ Res Public Health. 2022 Aug 17;19(16):10182. doi: 10.3390/ijerph191610182 (PMC9408527; doi:10.3390/ijerph191610182)
Supplement: Supplementary file 1 [file ijerph-19-10182-s001.zip › Supplementary Files S3.pdf]

Supplementary Files S3

Lexical-syntactic frames

**Sentence fragments (examples)**	**Lexical-syntactic frames**
Topic 1 Language Communication	
„can't understand"	VNeg ++ LangComContV
„language problem"	LangComN ++ EventNegN
„no one understood"	NegPro + LangComV
„difficulty in communication"	EventNegN ++ Prep ++ LangComN
„difficult to communicate"	NegAdj ++ Prep ++ LangComV
„problem is communication"	EventNegN ++ BeV ++ LangComN
„problem was about language"	EventNegN ++ BeV ++ Prep ++ LangComN
"lack of communication"	lack ++ Prep ++ LangComN
"doesn't know any other language than" Polish	VNeg ++ KnowV + any + LangComN + than
„don't know English"	VNeg ++ KnowV ++ LangComN
„nobody know English"	NegPro ++ KnowV ++ LangComN
„they didn't usually use English"	No specified frame/ rule
Topic 2 Type of medical service	
…he had some "infection" with his lower jaw and teeth because of lack of minerals….	RULE 1<br><br>If tokens of PatientIllness word group found, text classified as Illness
Resp mentioned in one story an episode of being "sick" and of getting medical checkup required for work	Classified as Illness by rule [ambiguous classification]
No tokens were searched for	RULE 2 (DEFAULT)<br><br>If no tokens of PatientIllness word group found, text classified by default as Checkup
„low blood pressure"<br><br>PatentIllness word group does not contain words "blood" or "illness", a correct identification would require 3-word phrase	Classified as Checkup by default rule [incorrect classification]
… "I have one problem related to my health but how I am not going to write. Having the problem I went to the hospital…"	Classified as Checkup by default rule [incorrect classification]

<table>
<tr><td colspan="2"><u>Topic 3 Health service personnel</u></td></tr>
<tr><td>“Next day we reached at clinic and consult to the doctor”</td><td>If tokens of MedPersonDocN word group found, classification as participation of physicians in a health service encounter</td></tr>
<tr><td>… “but the problem was receptionist couldn't speak English and I can't speak Polish”</td><td>If tokens of MedPersonOtherN word group found, classification as reference to participation of other, nonphysician personnel (medical or administrative) in a health service encounter</td></tr>
<tr><td>“people” as reference to health service personnel</td><td>Classified by default as no medical personnel participation [incorrect classification]</td></tr>
<tr><td>“one lady” as reference to medical personnel</td><td>Classified by default as no medical personnel participation [incorrect classification]</td></tr>
<tr><td>“they” as reference to ambulance personnel,

“he”, “him” as reference to a clinic personnel</td><td>Classified by default as no medical personnel participation [incorrect classification]</td></tr>
<tr><td>mention of “ambulance”, “hospital”, “emergency service” and no mention of medical personnel</td><td>Classified by default as no medical personnel participation [incorrect classification]</td></tr>
<tr><td colspan="2"><u>Topic 4 Change of health care unit</u></td></tr>
<tr><td>“Next day we reached at clinic and consult to the doctor”</td><td>If tokens of MedPersonDocN word group found, classification as participation of physicians in a health service encounter</td></tr>
<tr><td>… “but the problem was receptionist couldn't speak English and I can't speak Polish”</td><td>If tokens of MedPersonOtherN word group found, classification as reference to participation of other, nonphysician personnel (medical or administrative) in a health service encounter</td></tr>
<tr><td>“people” as reference to health service personnel</td><td>Classified by default as no medical personnel participation [incorrect classification]</td></tr>
<tr><td>“one lady” as reference to medical personnel</td><td>Classified by default as no medical personnel participation [incorrect classification]</td></tr>
<tr><td>“they” as reference to ambulance personnel,

“he”, “him” as reference to a clinic personnel</td><td>Classified by default as no medical personnel participation [incorrect classification]</td></tr>
<tr><td>mention of “ambulance”, “hospital”, “emergency service” and no mention of medical personnel</td><td>Classified by default as no medical personnel participation [incorrect classification]</td></tr>
</table>
