# Supplementary material for: Evaluating Patients’ Experiences with Healthcare Services: Extracting Domain and Language-Specific Information from Free-Text Narratives
Source: Int J Environ Res Public Health. 2022 Aug 17;19(16):10182. doi: 10.3390/ijerph191610182 (PMC9408527; doi:10.3390/ijerph191610182)
Supplement: Supplementary file 1 [file ijerph-19-10182-s001.zip › Supplementary Files S1.pdf]

Supplementary Files S1

Wordform types and tokens

Wordform	Number of tokens
and	571
the	493
i	470
to	447
was	299
my	298
in	257
hospital	209
he	186
for	184
of	184
we	183
a	180
so	177
doctor	170
that	164
is	146
they	123
but	118
very	118
me	117
with	111
it	107
after	101
medical	95
had	95
one	94
friend	93
problem	86
good	86
poland	86
some	85
have	78
she	75
not	73
went	72
time	70
about	64
got	64
this	63
all	61

because	61
from	61
treatment	60
there	60
are	60
english	59
t	57
when	56
experience	56
at	54
go	52
pain	52
then	50
on	49
language	48
test	48
insurance	46
am	46
her	46
him	45
us	45
as	44
health	44
an	44
his	43
xxx	43
by	42
day	40
checkup	39
you	38
also	38
first	37
appointment	37
take	36
took	36
medicine	36
gave	35
know	34
well	33
days	33
give	32
s	32
which	32
understand	31
or	31
were	30
like	30
body	29
get	29

doctors	27
don	26
here	26
job	25
blood	25
came	25
polish	25
no	25
our	25
where	25
fever	25
care	24
check	24
can	23
told	23
student	23
did	22
next	22
clinic	22
help	22
month	21
2	21
has	21
week	21
story	21
months	20
ago	20
visit	20
report	20
speak	20
who	19
asked	19
suffering	19
services	19
much	18
two	18
just	18
other	17
bad	17
how	17
cold	17
more	17
do	17
going	17
due	17
before	16
only	16
everything	16
want	16

money	16
patient	16
back	16
emergency	16
checked	15
come	15
near	15
up	15
really	15
country	15
last	15
service	15
meet	15
having	15
done	14
decided	14
given	14
any	14
many	14
healthcare	13
work	13
sick	13
another	13
now	13
home	13
be	13
helpful	13
staff	13
reception	13
even	13
communicate	13
feel	13
what	12
thing	12
visited	12
few	12
3	12
too	12
said	12
didn	12
people	12
problems	12
if	12
tell	12
stool	12
need	12
medicines	12
system	12
face	11

finally	11
talk	11
called	11
will	11
leg	11
lot	11
gone	11
pay	11
warsaw	11
ask	10
high	10
reach	10
injury	10
different	10
normal	10
friends	10
communication	10
company	10
foreign	10
would	10
started	10
satisfied	10
name	10
paid	10
call	10
been	10
used	10
brother	10
centrum	10
consult	9
process	9
share	9
close	9
india	9
stomach	9
right	9
faced	9
information	9
roommate	9
response	9
government	9
reports	9
them	9
overall	9
nearby	9
date	9
room	9
during	9
through	9

later	9
their	8
say	8
cost	8
found	8
tests	8
reached	8
main	8
condition	8
felt	8
getting	8
easy	8
free	8
wait	8
again	8
most	8
hour	8
situation	8
your	8
feeling	8
relief	8
accident	8
7	7
down	7
hand	7
long	7
taking	7
out	7
met	7
suggested	7
working	7
collect	7
taken	7
person	7
provided	7
receptionist	7
completed	7
consultation	7
head	7
totally	7
private	7
operation	7
weeks	7
zloty	7
google	7
5	7
using	7
food	7
address	7

indian	7
start	6
happened	6
show	6
basic	6
every	6
lack	6
environment	6
explained	6
examination	6
little	6
firstly	6
treated	6
number	6
apteka	6
whole	6
proper	6
tube	6
2018	6
its	6
suffered	6
than	6
while	6
same	6
best	6
hostel	6
disease	6
properly	6
ambulance	6
restaurantmc	6
urine	5
book	5
stuff	5
treat	5
testing	5
find	5
results	5
nice	5
way	5
procedure	5
seeking	5
weather	5
easily	5
lots	5
better	5
should	5
caring	5
issue	5
ear	5

stone	5
kidney	5
hours	5
rest	5
bit	5
part	5
giving	5
able	5
translate	5
laboratory	5
directly	5
covered	5
speaking	5
coming	5
night	5
4	5
ok	5
left	5
hospitals	5
over	5
skin	5
happen	5
infection	5
pln	5
fast	5
once	5
why	5
clinicat	5
use	5
suffer	5
translator	5
documents	5
dr	5
october	5
weight	4
manage	4
phone	4
fall	4
injured	4
broken	4
something	4
manager	4
advice	4
house	4
big	4
visa	4
token	4
amount	4
tomorrow	4

leave	4
unfortunately	4
end	4
patients	4
physician	4
required	4
details	4
clock	4
temperature	4
case	4
available	4
such	4
things	4
teeth	4
three	4
tried	4
listen	4
without	4
entered	4
explain	4
helped	4
clinicme	4
kind	4
minor	4
fees	4
1	4
morning	4
sample	4
card	4
year	4
warszawa	4
see	4
general	4
booked	4
severe	4
difficult	4
examined	4
living	4
soon	4
thank	4
eu	4
may	4
ill	4
made	4
reason	4
hello	4
actually	4
pressure	4
buy	4

both	4
anything	4
sister	4
small	4
suggest	4
question	4
around	4
sname	4
great	4
department	4
clinic	4
specialist	4
carefully	4
try	4
couldn	4
knee	4
located	4
chest	4
collected	4
college	4
smartphone	4
those	4
maybe	3
8	3
purpose	3
height	3
nurses	3
incident	3
bleeding	3
waiting	3
searched	3
restaurant	3
saying	3
new	3
procedures	3
each	3
result	3
university	3
normally	3
make	3
along	3
whom	3
fill	3
eye	3
followed	3
hot	3
somehow	3
students	3
cannot	3

lady	3
certificate	3
support	3
door	3
could	3
prescription	3
guide	3
suddenly	3
side	3
45	3
medicals	3
provide	3
drink	3
type	3
major	3
fit	3
pretty	3
guys	3
beginning	3
prescribed	3
cure	3
social	3
though	3
bill	3
under	3
own	3
o	3
moment	3
life	3
sleep	3
bed	3
understanding	3
staying	3
waited	3
attention	3
however	3
medication	3
pleasant	3
these	3
center	3
turn	3
regular	3
surprised	3
related	3
ever	3
viral	3
malaria	3
cousin	3
think	3

half	3
examine	3
wrong	3
heart	3
universityws	3
seen	3
stay	3
city	3
painful	3
thinking	3
eat	3
decide	3
informed	3
completely	3
seriously	3
worked	3
cover	3
cash	3
antibiotic	3
asking	3
500	3
mg	3
old	3
international	3
rain	3
second	3
doing	3
jack	3
antibiotics	3
road	3
expenses	3
scooter	3
personal	3
gives	3
suggestions	3
poor	3
minutes	3
apart	3
nurse	2
anyhow	2
place	2
everybody	2
knowledge	2
handled	2
anyone	2
queue	2
talked	2
further	2
previous	2

document	2
accept	2
quiet	2
makes	2
visitors	2
specific	2
rooms	2
consulted	2
perform	2
instructions	2
making	2
received	2
recommended	2
rules	2
follow	2
sure	2
passport	2
successfully	2
warm	2
climate	2
adjust	2
weakness	2
luckily	2
facilities	2
winter	2
today	2
luck	2
scared	2
handle	2
finished	2
happy	2
registered	2
doesn	2
change	2
since	2
enter	2
taxi	2
ward	2
cracked	2
wrist	2
conversation	2
form	2
payment	2
fee	2
bakery	2
complete	2
supposed	2
hala	2
matter	2

water	2
course	2
diagnosed	2
primary	2
arrived	2
guided	2
examinations	2
accompanied	2
current	2
thoroughly	2
writing	2
slipped	2
wet	2
floor	2
enough	2
powerful	2
security	2
vary	2
countries	2
might	2
arm	2
hurt	2
insurer	2
claim	2
whether	2
quite	2
tablets	2
years	2
0	2
low	2
nfz	2
delivery	2
hospitalba	2
realized	2
important	2
cough	2
completing	2
barrier	2
dear	2
hereby	2
attended	2
helpless	2
till	2
nothing	2
turned	2
worse	2
mostly	2
short	2
hospitality	2

submit	2
office	2
organized	2
inside	2
passed	2
study	2
together	2
headache	2
vomiting	2
level	2
routine	2
goes	2
times	2
supportive	2
else	2
rsurname	2
june	2
hospitalmi	2
dname	2
control	2
into	2
julia	2
shifted	2
recovering	2
breath	2
real	2
positive	2
mam	2
eyes	2
glucose	2
joint	2
disappointed	2
dislocated	2
respect	2
move	2
ochota	2
10	2
always	2
bottles	2
suggestion	2
starting	2
point	2
broke	2
fix	2
remember	2
charged	2
busy	2
public	2
trouble	2

charges	2
paying	2
fine	2
already	2
nepal	2
tumor	2
neck	2
shocked	2
healthy	2
sleeping	2
send	2
jaw	2
tonsil	2
250	2
slip	2
store	2
continuously	2
superb	2
kindly	2
friendly	2
local	2
6	2
full	2
searching	2
everyone	2
exercise	2
poisoning	2
cured	2
27th	2
september	2
search	2
etc	2
15	2
kebab	2
ache	2
recovered	2
slippery	2
foot	2
solve	2
improvement	2
car	2
driving	2
domino	2
fell	2
aid	2
satisfactory	2
charge	2
usually	2
tips	2

meeting	2
perfect	2
serious	2
knowing	2
walk	2
sufficient	2
guy	2
affected	2
dangerous	2
pick	2
consultancy	2
centre	2
someone	2
area	2
critical	2
boil	2
resturantkf	2
helping	2
mri	2
bump	2
pimple	2
generally	2
spend	2
worst	2
migraine	2
badly	1
documentation	1
forms	1
colleagues	1
run	1
seeing	1
girls	1
muttered	1
paper	1
father	1
replied	1
costly	1
hands	1
elbow	1
stitch	1
missing	1
six	1
undergone	1
prior	1
welcoming	1
appropriate	1
systemic	1
facilitates	1
certified	1

techniques	1
ample	1
performing	1
precisely	1
ease	1
finish	1
quickly	1
invited	1
performed	1
joined	1
initially	1
aware	1
regulations	1
least	1
approach	1
challenges	1
transportation	1
opposite	1
identification	1
proof	1
either	1
application	1
bmi	1
past	1
issues	1
compare	1
native	1
jet	1
lag	1
useful	1
recommend	1
facility	1
saturday	1
survived	1
august	1
direction	1
present	1
unbearable	1
managed	1
convince	1
earn	1
wanted	1
diagnose	1
hope	1
outside	1
delay	1
comfortable	1
difficulty	1
talking	1

obtained	1
stamp	1
medications	1
rid	1
expected	1
foreigners	1
eventually	1
payback	1
resp14	1
slept	1
snow	1
inject	1
hospitalso	1
xray	1
lucky	1
bones	1
ligaments	1
20	1
advantages	1
opened	1
accurate	1
furthermore	1
120	1
copy	1
gap	1
must	1
6th	1
march	1
clinicko	1
questions	1
mind	1
foremost	1
opinion	1
open	1
hence	1
tensed	1
criteria	1
internet	1
unsuccessful	1
definitely	1
exactly	1
advise	1
chronic	1
hernia	1
bother	1
angry	1
react	1
couple	1
ensure	1

organization	1
processing	1
flexible	1
proceed	1
alltogether	1
alone	1
indeed	1
convinced	1
painkillers	1
stage	1
surgery	1
accidentally	1
ground	1
words	1
wasn	1
stitches	1
systems	1
despite	1
business	1
trip	1
state	1
discovered	1
ehic	1
abroad	1
higher	1
knew	1
easier	1
pharmacist	1
satisfying	1
3rd	1
madam	1
checkups	1
duly	1
hi	1
pregnant	1
husband	1
select	1
sonography	1
admit	1
baby	1
born	1
knows	1
location	1
began	1
wake	1
min	1
ready	1
laying	1
dress	1

bus	1
although	1
list	1
sitting	1
concerned	1
expressing	1
roommates	1
concert	1
hospitalbu	1
szpital	1
held	1
seek	1
insisted	1
strongly	1
miserable	1
fainting	1
seems	1
rushed	1
ten	1
experiences	1
availing	1
comforting	1
initial	1
basis	1
eligibility	1
corporate	1
obviously	1
nervous	1
empty	1
inquired	1
settings	1
disciplined	1
eased	1
nervousness	1
introduction	1
comforted	1
formality	1
bp	1
signatures	1
submitted	1
newbie	1
physical	1
illness	1
fear	1
34	1
sugar	1
careful	1
feet	1
arriving	1

stayed	1
afraid	1
hospitalname	1
cream	1
technologies	1
nature	1
telling	1
mid	1
shivering	1
unable	1
contact	1
transfer	1
possible	1
infected	1
admited	1
total	1
1500	1
coverage	1
387	1
account	1
colleague	1
disorder	1
situated	1
accommodation	1
attend	1
read	1
cleanliness	1
active	1
excellent	1
man	1
tongue	1
bottle	1
poison	1
power	1
wawer	1
prescribe	1
tablet	1
heal	1
mba	1
write	1
particular	1
become	1
rude	1
event	1
far	1
step	1
lungs	1
beat	1
pulse	1

yesterday	1
injection	1
lastly	1
ended	1
precautions	1
admission	1
atmosphere	1
c	1
never	1
less	1
impressed	1
customer	1
agent	1
cotton	1
stick	1
happens	1
temporary	1
int	1
drops	1
playing	1
volleyball	1
bone	1
god	1
speaker	1
humble	1
calm	1
screaming	1
examining	1
mdam	1
summer	1
ending	1
uh	1
steps	1
desperate	1
angle	1
wants	1
afford	1
tough	1
per	1
law	1
admittance	1
website	1
facing	1
prefer	1
dec	1
25th	1
2016	1
entering	1
territory	1

being	1
hospitalization	1
successful	1
kept	1
thats	1
refunded	1
apartment	1
hadn	1
touch	1
site	1
reviews	1
jozefow	1
confirmed	1
humor	1
lower	1
minerals	1
800	1
meetings	1
off	1
orthopedition	1
swollen	1
ultimately	1
polmed	1
twist	1
pained	1
planned	1
clinicmi	1
prescriptions	1
stores	1
won	1
kajki	1
closed	1
pesel	1
100	1
id	1
let	1
killers	1
aged	1
maximum	1
ran	1
towards	1
became	1
fortunately	1
presented	1
fastly	1
team	1
diet	1
plans	1
quick	1

decision	1
regarding	1
entire	1
kindness	1
join	1
group	1
cool	1
natural	1
personalities	1
nose	1
throat	1
experienced	1
age	1
personality	1
enelmed	1
paula	1
wonderful	1
plus	1
heavy	1
necessary	1
contract	1
map	1
destination	1
written	1
live	1
21st	1
century	1
solutions	1
153	1
counter	1
125	1
tubes	1
describe	1
listened	1
massage	1
straight	1
away	1
europe	1
net	1
collection	1
collecting	1
mouth	1
simple	1
smoke	1
alcohol	1
allergic	1
meat	1
himself	1
allergies	1

expense	1
hospitalized	1
saline	1
evening	1
discharged	1
late	1
technology	1
bike	1
deep	1
cut	1
return	1
wasted	1
frustrated	1
inconveniency	1
friendresp73	1
fantastic	1
license	1
salary	1
fitness	1
seeked	1
appendicitis	1
needed	1
clinicmii	1
ad	1
countryuz	1
riding	1
workplace	1
knees	1
forehead	1
somewhat	1
strong	1
compared	1
nominal	1
delivered	1
publicly	1
funded	1
narodowy	1
fundusz	1
zdrowia	1
citizens	1
meaning	1
employer	1
spouse	1
child	1
insured	1
knowledgeable	1
studying	1
thought	1
diabetes	1

cooperative	1
period	1
feedback	1
admitted	1
increasing	1
european	1
stared	1
conditions	1
grade	1
especially	1
dizziness	1
movement	1
recovery	1
according	1
diagnosis	1
preventive	1
technique	1
sickness	1
arthritis	1
pharmacy	1
continued	1
dose	1
nutshell	1
pizza	1
translation	1
bajaj	1
organizational	1
band	1
wrap	1
legs	1
cross	1
signals	1
add	1
cooperate	1
distant	1
immunity	1
self	1
wouldn	1
online	1
reputed	1
sputum	1
shows	1
microorganisms	1
caused	1
advised	1
consultant	1
professionals	1
beloved	1
lived	1

50	1
registration	1
price	1
conscious	1
clients	1
behave	1
cooperated	1
myself	1
smart	1
converting	1
priority	1
pass	1
landed	1
look	1
yellowish	1
jaundice	1
liver	1
introduced	1
xname	1
yname	1
giddy	1
unconscious	1
burdened	1
meanwhile	1
hepatitis	1
via	1
hard	1
means	1
providing	1
helper	1
kitchen	1
washing	1
dish	1
clean	1
table	1
killer	1
carry	1
terrible	1
orthopedic	1
300	1
recently	1
sorry	1
muscles	1
stretching	1
cuts	1
deeply	1
pm	1
alright	1
dressing	1

wound	1
400	1
awesome	1
thanks	1
diarrhea	1
receipts	1
visiting	1
order	1
vital	1
signs	1
symptoms	1
analgesics	1
pleased	1
mother	1
nowadays	1
snowfall	1
impress	1
yes	1
understood	1
automatically	1
ticket	1
consuming	1
seat	1
consolation	1
building	1
assistant	1
cabin	1
allow	1
bench	1
dislike	1
bring	1
choice	1
prime	1
neurological	1
inform	1
ppo	1
direct	1
appreciative	1
shared	1
letter	1
arranged	1
satisfaction	1
created	1
restaurantmc	1
checking	1
ecg	1
beats	1
sharing	1
extra	1

duration	1
spoiled	1
please	1
special	1
behaviors	1
nobody	1
hire	1
persons	1
conveyed	1
message	1
filled	1
moved	1
safe	1
attack	1
regularly	1
helps	1
smartphones	1
physically	1
pills	1
siedlce	1
cheap	1
ears	1
preferred	1
sunday	1
wrote	1
description	1
express	1
scare	1
responding	1
within	1
identify	1
vaccinize	1
